# Effectiveness of Gentle Human Touch for Pain Control During Examination for Retinopathy of Pre-maturity: A Randomized Controlled Trial

**DOI:** 10.3389/fped.2020.608378

**Published:** 2020-12-17

**Authors:** Yongping Sun, Jinghan Zhang, Xu Chen, Yang Yang, Jie Qiu, Ke-yu Lu, Rui Cheng

**Affiliations:** Department of Neonates, Children's Hospital of Nanjing Medical University, Nanjing, China

**Keywords:** retinopathy of pre-maturity, neonates, screening, touch intervention, pain

## Abstract

**Background:** Retinopathy of pre-maturity (ROP) is a disorder of the retinal blood vessels in pre-term infants with low birth weight. It is a leading cause of blindness in children. During ROP screening, the use of mydriatic drops and eyelid openers causes pain and discomfort. Pain management strategies include medications and behavioral interventions. The objectives of this study was to investigate the effects of Gentle Human Touch on pain in pre-term infants undergoing screening for ROP.

**Methods:** In this randomized controlled trial, 82 infants in the neonatal intensive care unit at Children's Hospital of Nanjing Medical University who met the ROP screening criteria were randomly assigned to experimental and control groups using the random number table. The infants in the experimental group continuously received Gentle Human Touch during screening, while those in the control group were screened according to the routine procedure. All neonates were administered local eye anesthesia before the screening. The degree of pain was assessed using the Pre-mature Infant Pain Profile score. A double-channel near-infrared spectroscopy device was used to monitor regional cerebral oxygen saturation (rScO_2_), while oxygen saturation (SaO_2_) and heart rate were measured using pulse oximetry. The Pre-mature Infant Pain Profile score was the primary outcome, while heart rate, SaO_2_, and rScO_2_ were the secondary outcomes.

**Results:** The gestational age, corrected gestational age, birth weight, and Apgar score at examination and the basal heart rate, SaO_2_, and rScO_2_ showed no significant intergroup differences (*P* > 0.05 for all). Both groups demonstrated significant decreases in SaO_2_ and rScO_2_ in response to the examination (*P* < 0.05 for all). During the examination, the Pre-mature Infant Pain Profile score (14.82 ± 3.22 vs. 9.29 ± 2.89, respectively; *P* < 0.05) was significantly higher in the control group than in the experimental group, while rScO_2_ (57.61 ± 3.51 vs. 54.76 ± 4.54%, respectively; *P* < 0.05) and SaO_2_ (91.89 ± 6.43 vs. 85.68 ± 8.31%; *P* < 0.05) were significantly higher in the experimental group than in the control group. There was no significant difference in heart rate changes between the two groups before and after the examination (182.60 ± 3.50 vs. 170.80 ± 3.50 time/min; *P* > 0.05).

**Conclusions:** The findings of this study suggest that Gentle Human Touch can effectively alleviate pain during ROP screening in pre-mature infants.

**Clinical Trial Registration:** ISRCTN10976481, Registered 06 March 2020, Retrospectively registered.

## Introduction

Previously, there was a lack of awareness regarding neonatal pain, and no adequate analgesic measures were adopted for these patients, including those who were critically in need of repeated examinations (1). In fact, recurrent acute pain caused by invasive procedures induces a severe stress response in children (2). Excessive stress can increase central nervous sensitization in newborns, cause spinal axon remodeling, affect brain development, and result in chronic pain syndrome and physical discomfort in addition to stunted growth (3). Long-term effects include behavioral problems such as inattention and learning difficulties in childhood (4).

Retinopathy of pre-maturity (ROP) is one of the most common disease in pre-term infants (5). The incidence of ROP in neonatal intensive care units is high (6). It is characterized by abnormal proliferation of retinal blood vessels in pre-mature infants with low birth weight (7). There may be growth of abnormal blood vessels or damage and scarring of existing blood vessels in the retina. This scarring and bleeding can lead to retinal scarring or detachment from the back of the eye, resulting in vision loss. A standardized ROP screening procedure for pre-term infants is an effective way to prevent blindness (8). However, the procedures employed during ROP screening and treatment, such as the use of eyelid openers, scleral compression devices, mydriatic drugs, and binocular indirect ophthalmoscopy, can cause pain and discomfort (9). Therefore, it is important to relieve the pain of pre-mature infants caused by ROP screening.

Pain management strategies include medications and behavioral interventions. Because of the side effects of drugs, non-drug analgesic methods such as skin contact between the mother and child, touching, breastfeeding, feeding sugar, and non-nutritive sucking are commonly used (10). At home and abroad, several studies have evaluated the analgesic effects of sucrose or breast milk during ROP screening (11). However, to our knowledge, there is no report on the effects of Gentle Human Touch on the pain response during ROP screening. Therefore, to explore a convenient and effective intervention to alleviate pain, we conducted this randomized controlled trial (RCT) on the effects of continuous Gentle Human Touch on pain during ROP screening in pre-mature infants.

## Methods

### Trial Design

This prospective RCT was conducted in the tertiary level neonatal intensive care unit at Children's Hospital of Nanjing Medical University from January 1, 2018 to April 30, 2019. The main purpose of the preliminary study was to refine the research methodology and obtain sufficient data to carry out analysis of extended research. Based on the pilot trial with 20 patients in each group to obtain a 90% study power and a 5% significance level, a sample size of 25 was determined to be sufficient for this purpose. The initial sample size was calculated to detect a mean difference of 5 in the PIPP score between both groups. Considering that there may be data shedding, elimination, and poor patient compliance in the trial, we appropriately increased the number of cases based on the estimated sample size. This study was initiated with 86 pre-term infants; however, 4 pre-term infants were excluded due to the use of sedatives. Using the random number table, we assigned 41 pre-term infants to each group. The procedures followed in this study complied with the ethical standards set by the Ethics Committee of Children's Hospital of Nanjing Medical University and received approval from the committee. The study conformed to the standards set by the Declaration of Helsinki and Good Clinical Practice guidelines. The guardians of all children provided written informed consent for study participation and data publication.

### Participants

Pre-mature infants who met the following inclusion criteria based on the Guidelines for the Screening of Retinopathy of Pre-maturity in China issued by the Chinese Medical Association Ophthalmology Branch were considered eligible: pre-mature birth with a gestational age of ≤34 weeks or a birth weight of ≤2,000 g, no prior history of fundus screening, and screening at 4–6 weeks of age after birth or a corrected gestational age of 31–32 weeks. The exclusion criteria were as follows: administration of non-steroidal anti-inflammatory drugs or sedative and anti-epileptic drugs such as chloral hydrate, phenobarbital, and diazepam within 24 h before ROP screening; intolerance to screening because of critical conditions such as severe respiratory diseases, central nervous system infections, sepsis, and other organic diseases such as severe congenital heart malformation and pulmonary insufficiency. In this study, infants were assessed in clinically stable condition: awake, supine, and self-ventilating in air. The characteristics of the infants were recorded, including gestational age, corrected gestational age, birth weight, gender, and apgar scores.

### ROP Screening

The study personnel included two pediatric ophthalmologists with similar practice styles. All babies were examined in a similar fashion. First, their eyes were dilated using tropicamide (0.5%) and phenylephrine HCl (2.5%) before screening, and the dilation was repeated once every 10 min for a total of three times. A routine practice to reduce pain during the ROP examination was to swaddle the infant and administer one drop of a local analgesic (oxybuprocaine, 0.4%) immediately before the procedure. Retinal photos were acquired in the following order: posterior pole optic disc, macula, and temporal, upper, nasal, and lower quadrants. The procedure lasted for ~2 min.

### PIPP

The degree of pain before and during the examination were quantified using the Pre-mature Infant Pain Profile (PIPP) (12). The PIPP assessment items include the gestational age, increased HR, decreased blood oxygen saturation (SaO_2_), a state of arousal, and the proportion of painful expressions throughout the examination (frowning, blinking, and wrinkling the nose and sulcus). There were a total of seven items, each of which was scored on a scale of 0–3. The total PIPP score is calculated as the sum of the scores for all seven items, and the maximum score is 21 points. A higher score indicates greater pain severity. For calculation of the PIPP score, the infant's facial expressions and pulse oximetry findings were recorded throughout the procedure. The PIPP has been tested for reliability, validity and clinical utility with good results by Bonnie Stevens in 2010 (13).

### Near-Infrared Spectroscopy (NIRS)

An EGOS-600A NIRS meter (EnginMed, Suzhou, China) was used to collect data for the regional cerebral oxygen saturation (rScO_2_) before and during screening. Before screening, the NIRS probe was placed at the center of the forehead. The recorder traced a 2-min curve in the quiet state, and the stable value displayed after the curve represented the oxygen saturation of the basal brain tissue, which was then compared with the tissue oxygen saturation recorded at the time of pain during ROP screening. The tracing was repeated three times for each patient before and during examination. The average of the three results was used as the real-time rScO_2._ At the same time, a pulse oximeter (Comen Medical Instruments, Shenzhen, China) was used to measure SaO_2_ and HR.

### Gentle Human Touch

The Gentle Human Touch protocol (14) was implemented from the beginning of each procedure until 10 min after the procedure. The anesthesiologists placed the fingertips of the left hand above the eyebrow line with the palm touching the pre-term infant's crown. The rest of right hand and fingers rested on the infant's upper arm, while the right thumb was on the infant's right shoulder (midline position).

### Procedures

The research team comprised two research anesthesiologists, two child health care experts, and three assistants with extensive research and clinical experience. Following the acquisition of informed consent from the guardians, the infants were randomly assigned to either the experimental group or the control group using the random number table (the experimental group = 41, the control group = 41). The pre-term infants in the experimental group continuously received Gentle Human Touch during ROP screening, while those in the control group were screened according to the routine procedure. The ROP examinations were performed in a separate place in the NICU. The anesthesiologists gave GHT to infants in the experimental group at the beginning of the examinations. One of the researchers recorded the physiological measurements (heart rate, oxygen saturation, and regional cerebral oxygen saturation) of the pre-term infants on the Procedure Monitoring Form and scored the PIPP 5 min before and 5 min after the beginning of the ROP examination. Another researcher video recorded the ROP examinations, which lasted on 5 min. Three independent specialist the observed video records of the ROP examination and determined the PIPP scores. Then, inter-observer consistency analysis was performed for each item of the PIPP, which ranged between 0.90 and 1.00.

### Statistical Analysis

Data were expressed as percentage, arithmetic mean, standard deviation, median, minimum, and maximum. Differences in data between the two groups were tested using the unpaired *t*-test (normally distributed data), the Mann–Whitney *U*-test (non-normally distributed data). Comparisons of categorical variables were performed with Chi-square test or Fisher's exact test. Data comparison at different time points in the same group used the paired *t*-test. Analysis of variance using a two-factor repeated measures design to compare treatment, time, and the interaction between treatment and time. The inter-observer consistency among the three specialists evaluating the video records of the ROP examination used the intra-class correlation coefficient (ICC). Differences were considered significant if *p* < 0.05. Data were analyzed using SPSS version 24 (IBM Co., Armonk, NY, USA).

## Results

**Figure 1:**
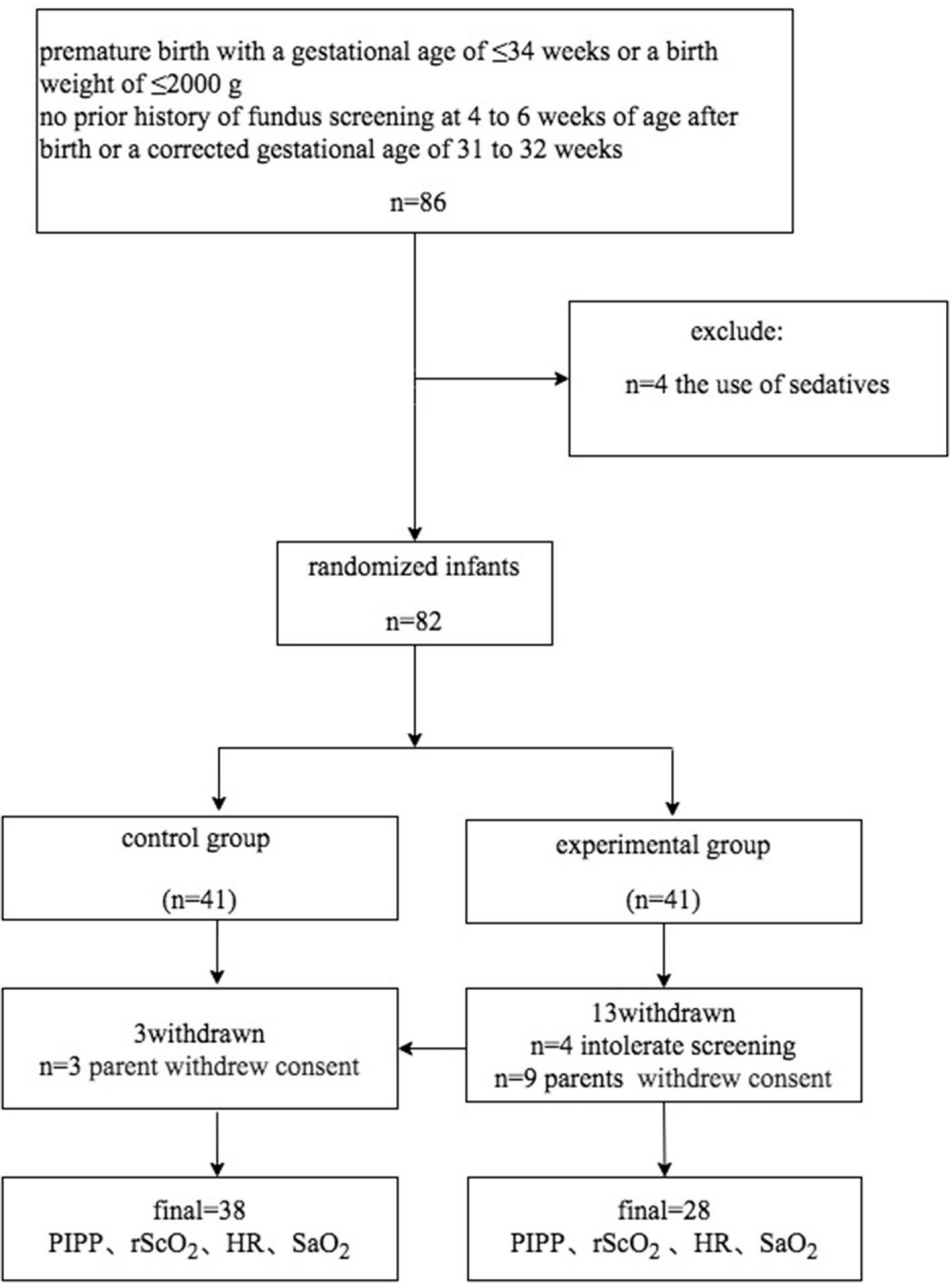
Flowchart showing patient inclusion into the randomized controlled trial on the effectiveness of touch intervention for pain control during screening for retinopathy of pre-maturity.

During the study period (January 1, 2018–April 30, 2019), 82 of 86 eligible infants were enrolled (Figure 1). Using the random number table, we assigned 41 pre-term infants to each group. 12 pre-term infants (three in the control group, nine in the experimental group) were excluded due to the parents withdrew consent. In the experimental group, four pre-term infants withdrew due to intolerance of the examination Eventually, the data of 66 neonates were included in the final analysis, with 38 babies in the control group and 28 babies in the experimental group. There was no significant difference in the demographic characteristics of the infants between the two groups (*P* > 0.05; Table 1). At the same time, a stratified analysis on the gender, age, and weight of the infants was been made. There is no difference between the two groups of infants in terms of gender, age, and weight. All infants in both groups were intensively treated for 24 h after ROP screening (Table 2).

**Table 1:** Clinical and demographic characteristics of neonates screened for retinopathy of pre-maturity with (experimental group) or without (control group) touch intervention.

	**Control group *N* = 38**	**Experimental group *N* = 28**	***P*-value**
Gestational age (weeks)	29.88 ± 0.40	30.03 ± 0.40	0.27
Corrected gestational age (weeks)	33.34 ± 1.79	33.70 ± 1.18	0.28
Birth weight (kg)	1.34 ± 0.56	1.32 ± 0.59	0.74
Sex (male/female)	20/18	14/14	0.83
1-min Apgar score	8(1–10)	8(5–9)	0.11
5-min Apgar score	9(1–10)	9(9–10)	0.29

**Table 2:** The characteristics analysis on the gender, age, and weight of the pre-term infants.

	**Control group**		**Experimental group**			
**Characteristics**	***n***	**%**	***n***	**%**	***X**^**2**^*	***P***
**Gender**						
Male	20	52.6	14	50.0	0.045	0.83
Female	18	47.4	14	50.0		
**Gestational age(week)**						
<28	8	21.1	5	17.9	0.104	0.75
≥28	30	78.9	23	82.1		
**Corrected gestational age(week)**						
<34	21	55.3	14	50.0	0.179	0.67
≥34	17	44.7	14	50.0		
**Birth weight (g)**						
<1,000	8	26.7	3	10.7	1.241	0.27
≥1,000	30	73.3	25	89.3		

### PIPP

There were no significant between-group differences in the basal HR (*P* > 0.05) (Table 3). The reductions from baseline in PIPP in the control group (11.82 ± 3.47) was higher than in the experimental group (6.29 ± 2.98) (*P* < 0.001) (Table 4). The PIPP score was significantly higher in the control group (14.82 ± 3.22) than in the experimental group (9.29 ± 2.89) during ROP screening (*P* < 0.05) (Table 5).

**Table 3:** Comparison of PIPP baseline value between control group and experimental group.

**Group**	***N***	**Baseline**	**During examination**	**Baseline comparison**	
				***t***	***p***
Control group	38	3.00 ± 1.01	14.82 ± 3.22	0.000	1.00
Experimental	28	3.00 ± 0.90	9.29 ± 2.89		

**Table 4:** Compare the changes in PIPP before and during the examination and the reduction from baseline in PIPP between the two groups.

**Group**	***N***	**Reduction from baseline(Difference within group)**	**Intra-group comparison**		**Comparison between groups**	
			***t***	***p***	***t***	***p***
Control group	38	11.82 ± 3.47	−20.986	<0.001^*^	6.785	<0.001^*^
Experimental	28	6.29 ± 2.98	−12.290	<0.001^*^		

* *P < 0.05*.

**Table 5:** The heart rate, oxygen saturation, regional cerebral oxygen saturation and PIPP before and during examination in pre-terterm infants.

	**Control group**	**Experimental group**	***F*-value**	***P*-value**
Baseline heart rate, bpm	139.08 ± 12.13	143.50 ± 11.56	2.418	0.125
Maximum heart rate during examination	170.79 ± 22.18	182.64 ± 18.49		
Baseline oxygen saturation,%	97.16 ± 2.39	96.86 ± 2.42	16.106	<0.001^*^
Minimum oxygen saturation during examination	85.68 ± 8.31	91.89 ± 6.43		
Baseline regional cerebral oxygen saturation%	62.57 ± 3.34	62.54 ± 3.28	8.744	0.004^*^
Minimum regional cerebral oxygen saturation during examination	54.76 ± 4.54	57.61 ± 3.51		
Baseline PIPP	3.00 ± 1.01	3.00 ± 0.90	48.289	0.000^*^
Maximum PIPP during examination	14.82 ± 3.22	9.29 ± 2.89		

* *P < 0.05*.

### rScO_2_

Before ROP screening, rScO_2_ was 62.57 ± 3.34% in the control group and 62.54 ± 3.38% in the experimental group, with no significant difference between groups (*P* > 0.05) (Table 6). Both groups demonstrated significant decreases in rScO_2_ during the examination. In the control group, rScO_2_ was 62.57 ± 3.34% before the examination and 57.61 ± 3.51% during the examination. In the experimental group, rScO2 was 62.54 ± 3.38% before the examination and 54.76 ± 4.54% during the examination (*P* < 0.001 for all) (Table 7). The reductions from baseline in rScO2 in the control group (−9.94 ± 8.98) was higher than in the experimental group (−4.61 ± 5.23) (*P* = 0.007) (Table 7). During the examination, rScO2 was significantly higher in the experimental group than in the control group (57.61 ± 3.51 vs. 54.76 ± 4.54%, respectively; *P* < 0.05) (Table 5).

**Table 6:** Comparison of rScO_2_ baseline value between control group and experimental group.

**Group**	***N***	**Baseline**	**During examination**	**Baseline comparison**	
				***t***	***p***
Control group	38	62.57 ± 3.34	54.76 ± 4.54	0.042	0.97
Experimental	28	62.54 ± 3.28	57.61 ± 3.51		

**Table 7:** Compare the changes in rScO_2_ before and during the examination and the reduction from baseline in rScO_2_ between the two groups.

**Group**	***N***	**Reduction from baseline(Difference within group)**	**Intra-group comparison**		**Comparison between groups**	
			***t***	***p***	***t***	***p***
Control group	38	−9.94 ± 8.98	11.825	<0.001^*^	−2.807	0.007^*^
Experimental	28	−4.61 ± 5.23	9.851	<0.001^*^		

* *P < 0.05*.

### SaO_2_ and HR

There were no significant between-group differences in the basal HR and SaO_2_ (*P* > 0.05) (Tables 8, 9). Both groups demonstrated significant decreases in SaO_2_ during the examination. In the control group, SaO2 was 97.16 ± 2.39% before the examination and 85.68 ± 8.31% during the examination. In the experimental group, ScO2 was 96.86 ± 2.42% before he examination and 91.89 ± 6.43% during the examination (*P* < 0.001 for all) (Table 10). The reductions from baseline in SaO_2_ in the control group (−11.47 ± 7.38) was higher than in the experimental group (−4.96 ± 5.10) (*P* < 0.001) (Table 10). During ROP screening, SaO_2_ was significantly higher (91.89 ± 6.43 vs. 85.68 ± 8.31%; *P* < 0.05) in the experimental group than in the control group (Table 5). There was no significant difference in heart rate changes between the two groups before and after the examination(182.60 ± 3.50 vs. 170.80 ± 3.50 time/min; *P* > 0.05) (Tables 5, 11).

**Table 8:** Comparison of SaO_2_ baseline value between control group and experimental group.

**Group**	***N***	**Baseline**	**During examination**	**Baseline comparison**	
				***t***	***p***
Control group	38	97.16 ± 2.39	85.68 ± 8.31	0.503	0.617
Experimental	28	96.86 ± 2.42	91.89 ± 6.43		

**Table 9:** Comparison of HR baseline value between control group and experimental group.

**Group**	***N***	**Baseline**	**During examination**	**Baseline comparison**	
				***t***	***p***
Control group	38	139.08 ± 12.13	170.79 ± 22.18	−1.493	0.140
Experimental	28	143.50 ± 11.56	182.64 ± 18.49		

**Table 10:** Compare the changes in SaO_2_ before and during the examination and the reduction from baseline in SaO_2_ between the two groups.

**Group**	***N***	**Reduction from baseline(Difference within group)**	**Intra-group comparison**		**Comparison between groups**	
			***t***	***p***	***t***	***p***
Control group	38	−11.47 ± 7.38	8.181	<0.001^*^	−4.013	<0.001^*^
Experimental	28	−4.96 ± 5.10	7.344	<0.001^*^		

* *P < 0.05*.

**Table 11:** Compare the changes in HR before and during the examination and the reduction from baseline in HR between the two groups.

**Group**	***N***	**Reduction from baseline(Difference within group)**	**Intra-group comparison**		**Comparison between groups**	
			***t***	***p***	***t***	***p***
Control group	38	31.71 ± 19.15	−10.206	<0.001^*^	−1.56	0.125
Experimental	28	39.14 ± 19.24	−5.835	<0.001^*^		

* *P < 0.05*.

## Discussion

To our knowledge, this was the first randomized controlled clinical trial to report the use of Gentle Human Touch for newborns during examination for ROP. A total of 82 infants who underwent eye examination for ROP screening were included in this study. There were no significant differences in demographic data and basal vital signs between infants who received Gentle Human Touch (experimental group) and those who did not (control group) during ROP screening. Research reveals that pain caused by ROP can increase HR and significantly decrease SaO_2_ in pre-term infants. Previous studies have shown that sensory nerve endings appear on the surface of the body, and the fetus can feel painful stimulations at a gestational age of 22–29 weeks (15). In the early stage of development, nerve endings are distributed such that they overlap, and local high excitability may occur. Thus, even mild stimulation can result in an excessive pain response (16). The body's physiological response to the stress caused by pain involves increases in catecholamine levels in the systemic circulation, HR, blood pressure, and intracranial pressure. Pathological changes caused by pain in neonates include hypoxia, hypercapnia, acidosis, hyperglycemia, and pneumothorax. Painful stimuli caused by aggressive procedures can induce vagal reflexes, which can cause hypoxia and changes in the cerebral blood flow (17). This is consistent with the changes in neonatal HR, SaO_2_, and rScO_2_ during the ROP operation in this study. Concurrently, repeated painful stimulation can cause changes in pain nerve pathways, neuroendocrine function, and neurodevelopment in the peripheral nerves and spinal cord (18). In the later stages, changes in the pain state or pain threshold, anxiety, stress disorders, and attention deficit can occur. Repeated pain stimulation during the early development of the nervous system can lead to persistent behavioral loss and partial sensory loss in the sensory area of the brain in pre-mature infants (19). Therefore, prevention and alleviation of pain in newborns, particularly pre-mature babies, are important.

The Guide to Neonatal Pain indicates that analgesic treatment mainly includes environmental measures, non-pharmacological measures, and drug-based treatment (12). For any procedure in newborns, clinicians should try and operate in a quiet and relaxed environment with minimum noxious stimuli such as light and noise (8). Non-pharmacological measures such as Gentle Human Touch, oral glucose water, pacifiers, and breastfeeding can distract newborns and prevent pain from transmitting to the cerebral cortex, thus having obvious analgesic effects (20). Therefore, we designed an RCT to test if Gentle Human Touch can relieve pain during screening of pre-mature infants for ROP. There was no significant difference between the mean oxygen saturation and heart rate measurements before the examination of the pre-term infants in the two groups. The mean oxygen saturation measurements were lower and the heart rate measurements were higher during the ROP examination (*P* < 0.05). Previous studies have also reported a decrease in blood oxygen saturation and an increase in heart rate during the ROP examination (20). In the current study, the mean PIPP scores of the pre-term infants in the two groups during the examination were higher (Table 4) (*P* < 0.001). In the ROP examination, ophthalmologists place a speculum on the eyes, put pressure on the eyeball, which result in a long and painful nature. The brightness of the ophthalmoscope may aggravate pain in pre-term infants (21). Therefore, interventions to effectively relieve pain should also be taken for newborns, despite the use of local anesthesia of the eyeball (22). The pacifying effects of touch intervention during painful procedures such as heel stick and venipuncture in term and pre-term neonates have been clearly shown in multiple studies (23). Accordingly, this study selected GHT for pain relief intervention in pre-mature infants. The PIPP score during ROP screening were significantly lower while SaO_2_ and rScO_2_ were significantly higher in the experimental group than in the control group (Table 3) (*P* < 0.05). These results indicated that Gentle Human Touch significantly reduced the stress response to pain stimuli and effectively relieved pain in pre-mature infants being screened for ROP. Gentle Human Touch helps the body to reduce the stress response to pain and diminishes crying. Our previous study found that Gentle Human Touch combined with music therapy can reduce the pain response in pre-term infants undergoing tracheal intubation by significantly increasing β-endorphin levels rather than increasing blood cortisol levels (24). Moreover, it can promote the development of the nervous system, balance the development of the cerebellum and brain, and enhance the ability of newborns to resist pain and damage. Thus, it can have an important effect on the intellectual and psychological development in the initial life stages of the child.

A novel aspect of the present study is the use of NIRS for pain assessment during ROP screening. Previously, PIPP scores based on subjective judgment were used to evaluate pain in pre-term infants. The use of non-invasive NIRS to monitor changes in rScO_2_ is a more objective method (25). Recent studies have investigated emerging techniques for measuring pain responses, such as NIRS, amplitude-integrated electroencephalography, functional magnetic resonance imaging, skin conductance, and HR variability assessment (26). Among these, NIRS is a non-invasive technique that reflects changes in brain hemodynamics and monitors SaO_2_ in specific organs such as the brain, kidneys, and intestines, thus reflecting tissue perfusion and oxygen supply and demand. The parameter rScO_2_ is also known as the tissue oxygenation index, which reflects the state of tissue oxygen supply (27). Jean-Michel found either evaluate the changes in NIRS (differences between pre-venepuncture and post-venepuncture values) or evaluate the maximum NIRS values, to discriminate pain in full-term or pre-term neonates (28). To our knowledge, few studies have used NIRS to assess the effects of Gentle Human Touch on pain in pre-term infants. Our study found that rScO_2_ during ROP screening was significantly lower than the basal brain tissue oxygen saturation, although it was significantly higher in the experimental group than in the control group. Similar results were observed for SaO_2_. This suggests that NIRS can be used as an effective and safe method for monitoring pain in newborns.

Our study also has some limitations. First, the sample size was small, and future studies will need to include more children for further verification of our findings. Second, we only evaluated a baseline before the exam and score during the exam. The best way to evaluate will be to have a baseline before the exam, score during the exam and most importantly, evaluation of the infant's condition after the exam and the time it needs for the infant to return to pre-exam baseline. We will expand the sample size and add more details to continue to improve this experiment. In addition, after grouping, both groups had the study subjects withdraw, which could cause attrition bias in the trial. Although our patients who withdrew did not participate in the treatment and had no outcome data, it also could cause attrition bias. In the situation, the multiple filling model can be used for *post-hoc* analysis to study whether the attrition bias changes the results. We used PIPP to assess pain, subjective errors while assessing the distress of another individual are inevitable. Nevertheless, pain monitoring based on vital signs such as HR and SaO_2_ is simple, convenient, and feasible. Further studies on hormonal changes during pain responses in pre-term infants undergoing ROP screening are necessary to further clarify the pathological mechanism underlying the pain.

## Conclusions

In conclusion, the findings of this study suggest that Gentle Human Touch can effectively alleviate pain during ROP screening in pre-mature infants.

## Data Availability Statement

The original contributions generated for the study are included in the article/supplementary material, further inquiries can be directed to the corresponding authors.

## Ethics Statement

The studies involving human participants were reviewed and approved by the procedures followed in this study complied with the ethical standards set by the Ethics Committee of Children's Hospital of Nanjing Medical University and received approval from the committee. Written informed consent to participate in this study was provided by the participants' legal guardian/next of kin.

## Author Contributions

YS was an assistant of the research team, evaluated the PIPP score, and performed the statistical analysis. JZ was an assistant of the research team and measured oxygen saturation and heart rate. XC, YY, and JQ were the research neonatologists of the research team. RC developed the specific study idea, and as a neonatal expert guided the implementation of specific clinical experimental operations. K-yL participated in the design of the experiment and wrote the manuscript. All authors made enormous contributions to the design of this study and preparation of the manuscript.

## Conflict of Interest

The authors declare that the research was conducted in the absence of any commercial or financial relationships that could be construed as a potential conflict of interest.

## Abbreviations

ROP: retinopathy of pre-maturity
PIPP: Pre-mature Infant Pain Profile
NIRS: near-infrared spectroscopy
rScO_2_: regional cerebral oxygen saturation
HR: heart rate
SaO_2_: oxygen saturation
RCT: randomized controlled trial.
